# Severe reactive ischemic posterior segment inflammation in Acanthamoeba keratitis: Case report of a patient with Sjögren’s syndrome

**DOI:** 10.1007/s00717-017-0334-0

**Published:** 2017-03-09

**Authors:** Christoph Palme, Bernhard Steger, Gertrud Haas, Barbara Teuchner, Nikolaos E. Bechrakis

**Affiliations:** Department of Ophthalmology, Medical University of Innsbruck, Anichstraße 35, 6020 Innsbruck, Austria

**Keywords:** Acanthamoeba keratitis, Reactive ischemic posterior segment inflammation, Sjögren’s syndrome, Cataract surgery, Acanthamoeba-Keratitis, Reaktive ischämische Entzündung des hinteren Augenabschnitts, Sjögren-Syndrom, Kataraktoperation

## Abstract

**Purpose:**

We report on a case of Acanthamoeba keratitis (AK)-related reactive ischemic posterior segment inflammation following intraocular surgery in a patient with primary Sjögren’s syndrome (PSS).

**Case report:**

A 48-year-old female patient with severe protracted AK underwent uneventful cataract surgery upon development of a corneal scar. Four weeks postoperatively, she experienced a rapid loss of vision to no light perception. Central retinal artery occlusion and ischemic optic neuropathy could be excluded, and a diagnosis of PSS was made. The condition remained unresponsive to systemic steroid treatment and ultimately led to enucleation of the globe. Histologic work-up revealed ischemic posterior segment inflammation and Acanthamoeba cysts in the corneal stroma.

**Conclusion:**

Autoimmune disease may be a risk factor for AK-related severe reactive ischemic posterior segment inflammation, and intraocular surgery can be a trigger to its manifestation.

## Zusammenfassung

**Hintergrund:**

Es wird über den Fall einer durch eine Acanthamoeba-Keratitis (AK) induzierten reaktiven ischämischen Entzündung des hinteren Augenabschnitts nach intraokularer Operation bei einer Patientin mit primärem Sjögren-Syndrom (PSS) berichtet.

**Methodik:**

Bei einer 48-jährigen Patientin mit schwerer protrahierter AK wurde eine komplikationslose Kataraktoperation nach Entwicklung einer Hornhautnarbe durchgeführt. Vier Wochen postoperativ trat ein rapider Sehverlust bis zur fehlenden Lichtwahrnehmung auf. Nachdem ein Zentralarterienverschluss der Retina und eine ischämische Optikusneuropathie ausgeschlossen werden konnten, wurde die Diagnose eines PSS gestellt. Die Erkrankung sprach nicht auf eine systemische Steroidbehandlung an und führte schließlich zur Enukleation.

**Ergebnisse:**

Die histologische Aufarbeitung ergab eine ischämische Entzündung des hinteren Augenabschnitts und Acanthamoeba-Zysten im Hornhautstroma.

**Schlussfolgerung:**

Eine Autoimmunkrankheit stellt möglicherweise einen Risikofaktor für eine AK-bedingte schwere reaktive ischämische Entzündung des hinteren Augenabschnitts dar, und eine intraokulare Operation kann ein Auslöser für die klinische Manifestation sein.

## Introduction

Reactive ischemic posterior segment inflammation (RIPSI) has been reported as a rare sight-threatening manifestation of prolonged severe Acanthamoeba keratitis (AK) [1]. Occurring especially in patients with underlying hypercoagulation disorders, histopathologic correlates include chronic chorioretinal inflammation with perivascular lymphocytic infiltration and retinal vascular thrombosis. Primary Sjögren’s syndrome (PSS) is a chronic inflammatory autoimmune disorder that causes destruction of lacrimal and salivary glands [2]. Ischemic optic neuropathy and choroidopathy have been reported as sight-threatening manifestations of PSS [3, 4]. We report on a case of AK-related reactive ischemic posterior segment inflammation in a patient with this autoimmune disease.

## Case report

**Fig. 1:**
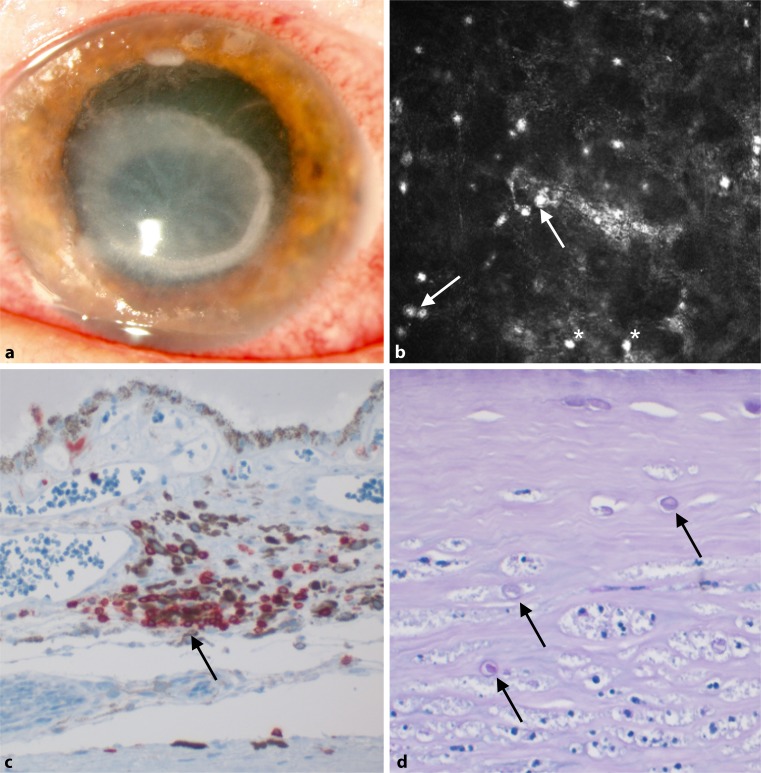
**a** Color photographic image at initial presentation showing a large central corneal ring infiltrate and a second smaller anterior stromal infiltrate in the superior peripheral cornea. **b** In vivo confocal microscopic image showing the presence of numerous double-walled Acanthamoeba cysts (*white arrows*) and presumed trophozoites (*asterisks*) in the central anterior corneal stroma. Field size 400 × 400 µm, focus depth set at 58 µm. **c** Photomicrograph of an immunohistochemical staining for CD45, showing dense choroidal lymphocytic infiltration in proximity to a choroidal vein. Original magnification, ×400. **d** Photomicrograph of the anterior cornea demonstrating Acanthamoeba cysts embedded between stromal lamellae, ectocysts with retracted endocyst (*arrows*). Periodic acid-Schiff stain; original magnification, ×200

A 48-year-old female myopic patient presented with a 3-week history of progressive pain in her left eye. She had been wearing rigid gas permeable contact lenses and used self-prescribed antibiotic and steroid eye drops. Her past medical and ocular history was unremarkable. Initial best corrected visual acuity (BCVA) was counting fingers. Biomicroscopic examination revealed a large ring-shaped corneal stromal infiltrate, a central corneal epithelial defect in the absence of a hypopyon (Fig. 1a). In vivo confocal microscopy (IVCM) of the cornea was performed and confirmed the clinically suspected diagnosis of AK, showing a large number of Acanthamoeba cysts in the anterior stroma (Fig. 1b). Full ophthalmologic examination of the other eye was unremarkable apart from signs of moderate dry eye disease. The patient was hospitalized and treated with a topical antimicrobial treatment regimen consisting of half-hourly polyhexamethylene biguanide 0.02%, chlorhexidine 0.02%, and propamidine isethionate (0.1%). Additionally, topical treatment with tobramycin and fluconazole eye drops was initiated upon culture results from the contact lens positive for *Klebsiella pneumoniae*, *Pseudomonas aeruginosa,* and *Candida albicans*. Under this treatment the infiltrate improved significantly and the patient could be discharged with a BCVA of 0.80 on a LogMAR scale with ongoing topical treatment.

After 3 months and settled inflammation, the patient’s BCVA deteriorated to hand movements secondary to a white intumescent cataract with lens swelling. She underwent uncomplicated phacoemulsification surgery with implantation of a posterior chamber intraocular lens. Postoperative BCVA improved to 0.60 LogMAR. Four weeks postoperatively the patient experienced a rapid painless loss of vision to no perception of light. Clinical examination was remarkable for profound ocular hypotension with an intraocular pressure of 4 mm Hg. Optical coherence tomography (OCT) of the macula, although of reduced quality, showed hypotensive maculopathy with macular folds due to choroidal effusion, and mild optic disc edema. Fluorescence angiographic imaging of the retinal vasculature was attempted but remained inconclusive because of reduced fundus visibility. Optic neuritis and demyelinating disease of the central nervous system were ruled out by magnetic resonance imaging. No focal neurologic signs were present. Visual evoked potentials were attenuated and delayed in the affected eye. Further diagnostic work-up yielded positive results for anti-SSA (Ro) autoantibodies and increased levels of antinuclear antibodies, whereas anti-phospholipid antibodies and lupus anti-coagulant remained negative. Additionally, ultrasonographic examination of the parotid gland revealed evident parenchymal inhomogeneity verifying salivary gland involvement [5]. The patient confirmed symptoms of dry eyes, dry mouth, and relapsing arthritis and was diagnosed with PSS by the local rheumatology service meeting current diagnostic consensus criteria [6]. She was treated with prednisolone 250 mg and acetylsalicylic acid 100 mg once daily but did not regain any vision. Subsequently, she developed neuropathic corneal ulceration, band keratopathy in the setting of ocular hypotension, which ultimately necessitated enucleation of the eye. Histologic work-up revealed numerous Acanthamoeba cysts in the corneal stroma but no intraocular cysts. Intraocular findings included lymphocytic infiltrates in the choroidal stroma, around the choroidal veins and the short posterior ciliary arteries, diffuse retinal atrophy, but no evidence of retinal vascular thrombosis (Fig. 1c, d). No multinucleated perivascular cells were identified.

## Discussion

In the presented case, a patient with previously undiagnosed PSS developed AK-related severe reactive ischemic posterior segment inflammation (RIPSI) with a dismal outcome following intraocular surgery. AK-induced autoimmunity has previously been discussed as the underlying disease mechanism of retinal and choroidal vasculitis. RIPSI has been described as severe visual loss in patients with protracted and refractory AK. Histopathologic correlates include chorioretinal inflammation with perivascular lymphocytic infiltration and retinal vascular thrombosis, as present in our patient [1].

Hamrah et al. showed that in keratitis corneal antigen-presenting cells can migrate to the cervical lymph nodes and induce a state of autoimmunity through molecular mimicry. Activated T cells can in turn initiate a significant inflammatory reaction in the vascularized parts of the globe, mainly the uveal tissue and the retina [7]. Autoimmune ischemic choroidopathy likewise is a known complication of PSS, potentially precipitated by infection-related inflammatory response with induction of the interferon-1 (IFN-1) pathway [4, 8]. However, histopathologic examination of isolated PSS-related optic neuropathy would not be expected to show pronounced perivascular inflammatory cell infiltration in large choroidal vessels, as shown in Fig. 1, since it fits more accurately the previously described findings in RIPSI.

Furthermore, even uncomplicated intraocular surgery significantly increases intraocular and systemic levels of pro-inflammatory cytokines including IFN-1 [9]. Awwad et al. described an association with hypercoagulation disorders, but no patient was found to suffer from autoimmune disease. However, the authors discuss the possible induction of a state of autoimmunity through molecular mimicry via corneal antigen-presenting cells. A type III immune reaction could form and may target distal vascular tissue receptors leading to vasculitis and vascular thrombosis, ending with tissue necrosis [1]. In addition, our findings point toward an association between autoimmune disease and AK-related RIPSI, and intraocular surgery as a trigger to its manifestation.
